# MSK-TIM: A Telerobotic Ultrasound System for Assessing the Musculoskeletal System

**DOI:** 10.3390/s24072368

**Published:** 2024-04-08

**Authors:** Zachary Ochitwa, Reza Fotouhi, Scott J. Adams, Adriana Paola Noguera Cundar, Haron Obaid

**Affiliations:** 1 Department of Mechanical Engineering, University of Saskatchewan, Saskatoon, SK S7N 5A9, Canada; zachary.ochitwa@usask.ca (Z.O.); apn252@usask.ca (A.P.N.C.); 2 Department of Medical Imaging, University of Saskatchewan, Saskatoon, SK S7N 0W8, Canada; scott.adams@usask.ca (S.J.A.); haron.obaid@usask.ca (H.O.)

**Keywords:** telemedicine, telerobotic ultrasound, musculoskeletal, robotics

## Abstract

The aim of this paper is to investigate technological advancements made to a robotic tele-ultrasound system for musculoskeletal imaging, the MSK-TIM (Musculoskeletal Telerobotic Imaging Machine). The hardware was enhanced with a force feedback sensor and a new controller was introduced. Software improvements were developed which allowed the operator to access ultrasound functions such as focus, depth, gain, zoom, color, and power Doppler controls. The device was equipped with Wi-Fi network capability which allowed the master and slave stations to be positioned in different locations. A trial assessing the system to scan the wrist was conducted with twelve participants, for a total of twenty-four arms. Both the participants and radiologist reported their experience. The images obtained were determined to be of satisfactory quality for diagnosis. The system improvements resulted in a better user and patient experience for the radiologist and participants. Latency with the VPN configuration was similar to the WLAN in our experiments. This research explores several technologies in medical telerobotics and provides insight into how they should be used in future. This study provides evidence to support larger-scale trials of the MSK-TIM for musculoskeletal imaging.

## 1. Introduction

Robots have grown increasingly popular in the field of medicine. They have been shown to help both doctors and patients by improving working conditions and surgical outcomes [1,2]. One area of recent growth, especially with the recent pandemic, is the use of telemedicine [3]. Telemedicine is the practice of medicine, such as doctor’s appointments and assessments, over remote distances and often through phone or video conferences.

There are several advantages of providing clinical services remotely. The first and most significant is the improvement of accessibility. Patients living in remote or rural communities would no longer need to drive long distances to see medical practitioners with the necessary equipment or training. As a result, patients would require less travel time and incur fewer expenses while possibly getting a faster diagnosis [4]. A total of 44% of the world’s population and 18% of the Canadian population live in rural communities [5]. Thus, there is a large population that can see possible life improvements with the development of telemedicine. Telemedicine may also be used when traveling in vehicles such as ambulances or planes [6]. Another benefit is that limited contact can reduce the spread of infectious diseases.

One area of telemedicine is remote diagnostic ultrasound via robotics, also known as telerobotic ultrasound. Instead of the radiologist directly holding the probe to scan a patient, they use a remote manipulator to move the probe as they desire. An example of this is the MELODY system. This three-degree-of-freedom (DOF) manipulator mimics the motion of a “dummy probe” held by the operator [7]. Another commercially available alternative is Medirob which is a 6-DOF system controlled by a three-dimensional mouse [7]. Other systems developed by research groups include the dual-arm iFIND [8], ReMeDi with its mobile base [9], and the 4-DOF Estele [10]. These devices were designed for abdominal imaging and allow for remote or long-distance examinations. Besides improving accessibility, robotic tele-ultrasound can improve the ergonomic conditions of the radiologist and reduce the risk of repetitive stress injuries [11,12]. It can also improve position accuracy and reduce tremors while using the probe [13].

While there are several benefits of conducting ultrasound imaging remotely, there are also challenges. The first is the loss of tactile information when operating remotely. In conventional ultrasound, the radiologist holds the ultrasound probe in their hands and can feel the pressure applied to the patient, using it to palpate or feel the anatomy beneath the surface. However, this sense of touch is lost when the ultrasound probe is instead held by a robotic manipulator. This may result in a degraded experience.

Force feedback is used for multiple purposes in ultrasound. The first and most important application of force feedback is to ensure the safety of the patient. If a robot is unaware of its interaction with the human, it may cause accidental harm. Therefore, force measurement systems must be designed to help regulate contact and prevent harm. The use of force feedback has been shown to reduce the average and peak applied forces [14,15]. The second application of force feedback is to palpate the anatomy beneath the surface of the skin [16]. Finally, force feedback is used to ensure good image quality. An insufficient force may result in poor contact and air gaps between the probe and object being scanned. Excessive force may compress the tissue being imaged, making it difficult to interpret [17,18]. Due to its many applications, force feedback is an essential feature for robotic tele-ultrasound systems.

Therefore, it is important to develop force feedback for remote-operated systems. Recently, technical requirements have been proposed and a few devices have implemented haptic feedback. For example, ref. [19] proposes a communication layout and multiple layers of safety systems including software and hardware, and recommends force regulation with the motion of the patient, including breathing. The system in [20] automatically cut power from the manipulator once the measured contact force exceeded a safety threshold. The systems developed in [14,21] were successfully implemented in clinical trials.

The second major area of improvement is the time delay or latency in communicating between the operator and patient. Depending on the equipment, a time delay can be from 30 ms to 1.5 s [22,23]. Latency in the position control signal can cause undesired error in position control which can be a safety concern [24]. Investigating latency can help identify locations where delay can be minimized. Additionally, understanding the characteristics of latency can help inform design choices for a delay-mitigating control scheme. Some controllers such as the Bilateral Generalized Predictor Controller proposed in [25] used an estimated value of delay to predict what control signal would be appropriate considering the desired motion and the communication delay with the manipulator. If too large of an estimate delay is used, the predicted control signal would be less accurate. If the estimate delay is too small, the delay would have a larger effect on the system. Therefore, an informed choice of delay is beneficial in controller design. Previous work has measured a full system delay within a local network [26]. This study will investigate both the system and network delay in remote control over the internet.

To advance the field of teleoperated systems, we developed a telerobotic ultrasound manipulator designed for the musculoskeletal system called MSK-TIM (Musculoskeletal Telerobotic Imaging Machine). A predecessor to this system has been tested and shown to be feasibly used over a Wireless Local Area Network (WLAN) but had room for improvement. Previous trials to develop a musculoskeletal telerobotic machine had a few limitations such as lack of remote control of ultrasound functions, lack of force feedback, and lack of easy remote connectivity between slave and master stations [4,26]. Therefore, this paper describes research conducted to (1) investigate how adding visual force feedback affects patients’ comfort and experience, (2) study how using a wireless connection changes the delay time and quality of control, and (3) explore how introducing remote control of ultrasound functions can help optimize image quality.

## 2. Methodology

### 2.1. MSK-TIM System

The telerobotic ultrasound system used in this trial is a modified version of the musculoskeletal (MSK) telerobotic ultrasound device used in the previous trial [4]. Unlike many of the existing tele-ultrasound robots, which are designed for abdominal imaging, the MSK-TIM is designed for musculoskeletal imaging. It consists of a master station, a slave station, and a communication network.

**Figure 1:**
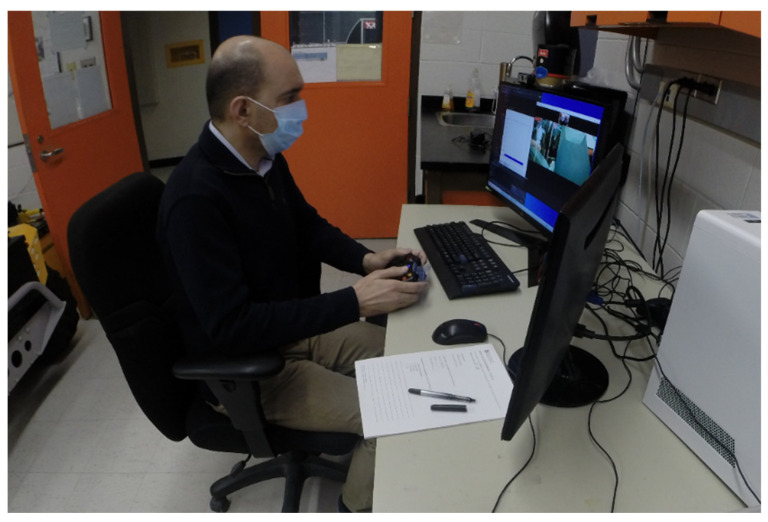
Setup of the master station for the musculoskeletal telesonography system.

The master station or the expert site, as seen in Figure 1, is responsible for controlling the manipulator, providing ultrasound imaging, and giving feedback to the operator. This station only requires a computer with a camera and controller. In our recent trial, a gamepad controller was used to move the manipulator. Video conferencing and remote desktop software were used to receive video from the slave station and change ultrasound settings remotely while a custom graphical user interface (GUI) was used to visually indicate the applied force.

**Figure 2:**
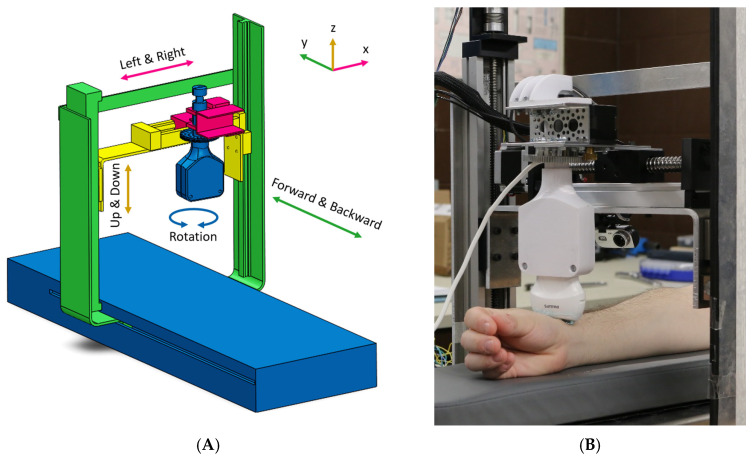
Schematic (**A**) and prototype (**B**) of the slave manipulator of the telesonography system.

The slave station (or the patient site) is responsible for moving the probe and collecting ultrasound images. The equipment at this station includes a robotic manipulator and a laptop with several cameras. The robot, seen in Figure 2A,B, is a 4-DOF manipulator with three prismatic joints and one revolute joint with an ultrasound probe at its end-effector. The *X*-axis is horizontal in the transverse direction, the *Y*-axis is horizontal along the length, and the *Z*-axis is in the vertical direction. The position of the participant’s arm is indicated with wide-view and close-up cameras.

**Figure 3:**
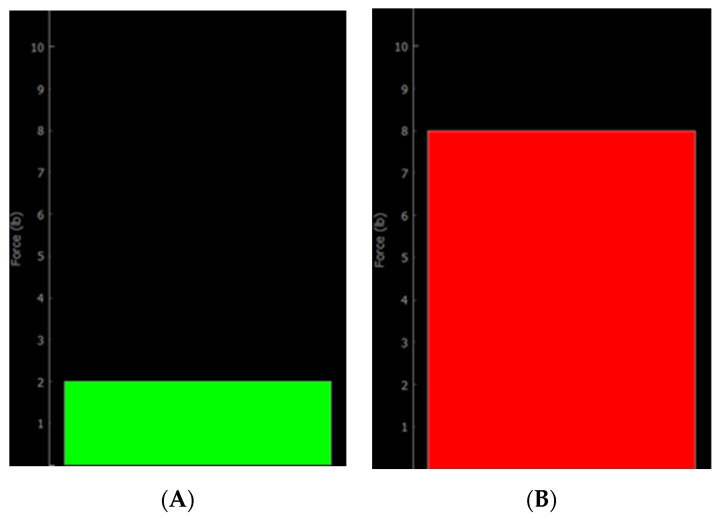
Force feedback graphic user interface (GUI) and low (**A**) and high (**B**) loads. Green indicates contact whereas red indicates a potentially excessive load.

One major upgrade to our system was the installation of a load cell. This load cell measures the force applied to the patient’s body in the vertical direction for safety and image quality. This 1-DOF compressive load cell transmits an analogue signal to a microcontroller on the slave side. The measured force is measured at a frequency of 30 Hz and transmitted to the master station where it is displayed as a live bar graph, as shown in Figure 3. Therefore, the bar graph is a type of visual force feedback which measures the vertical force applied to the patient’s arm. Both the magnitude and color of the bar graph change depending on the magnitude of the force, with green indicating contact, yellow indicating a moderate force, and red indicating a potentially excessive force [12]. In future development, the bar graph can be replaced with haptic feedback utilizing multiple degrees of freedom.

**Figure 4:**
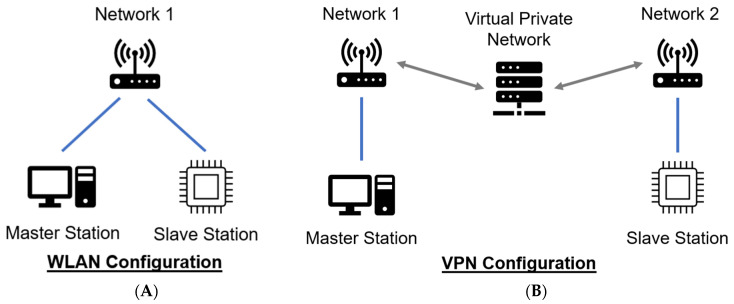
Experimental network configurations including the Wireless Local Area Network (WLAN) (**A**) and Virtual Private Network (VPN) (**B**) configuration.

Two different communication networks were used during the trial. The primary Wi-Fi network was measured at an average of 85 Mbps download and 82 Mbps upload speed. The second network, which was used to test the connection between the different networks, was measured at an average network speed of 168 Mbps download and 138 Mbps upload. The MSK-TIM system was upgraded so that the master and slave stations can communicate despite being on different networks using a virtual private network (VPN). Therefore, the two stations can be physically separated to two locations with internet access. To investigate how this change affects system latency, this study investigates both the VPN and the Wireless Local Area Network (WLAN) employed in previous studies [4,26]. An explanatory diagram can be seen in Figure 4. Future work includes implementing methods to mitigate the effects of delay.

The time delay in the system was measured using two methods: first, a timer program, and second, synchronized video recordings. The timer program measures the round-trip delay solely in the communication between the master and slave station with a resolution of less than 1 millisecond. This is done by measuring the time for the master station to send a package to the slave and receive a response back. The video recordings measure the unidirectional delay of the entire system, from the controller input in the master station to the resulting motion in the slave station. The measured value will be a summation of delays from sources such as the gamepad controller, network, software, and motors. The resolution of the video recordings is limited by the camera framerate which is 60 Hz or 16.7 ms. The timer program measures delay at a constant rate of 30 Hz while the video recording measures one delay value per controller input.

Two additional changes were made to the MSK-TIM device. The first was adding the ability to remotely configure ultrasound parameters including focus, depth, gain, zoom, color, and power Doppler controls. The radiologist adjusted the controls on the slave computer through remote desktop software. Secondly, the controller was changed from a joystick to a gamepad controller to improve ergonomics and ease-of-use for the radiologist. As the ease-of-use improves, the training and examination time should decrease.

### 2.2. Clinical Assessment

This clinical study was approved by the University of Saskatchewan Research Ethics Board and written informed consent was obtained from each participant. Telerobotic ultrasound exams were performed on the bilateral wrists of twelve participants (10 male, 2 female) or a total of twenty-four human arms. All participants were presumed to be healthy with no known MSK pathology. The operator was a musculoskeletal radiologist with 20 years of experience. This radiologist underwent a one-hour training session prior to the trial. The radiologist and participants were in separate rooms and could only communicate through video conferencing. Anisotropy was overcome by adjusting both the probe and extremity position. During the trial, the latency and overall duration of the exams were recorded, and ultrasound images were archived. The ultrasound images were visually assessed by two experienced radiologists in a separate session from the experimental trial.

After each exam, both the participant and the radiologist completed a survey to describe their experience [4]. The participant questionnaire included questions related to comfort, communication with the radiologist, and overall experience. Following the trial, the radiologist used a Likert scale from 1 to 5 (where 1 is inadequate and 5 is perfect) to assess communication with participants, image quality, ergonomics, and radiologist’s convenience. The radiologist and participants were given the opportunity to provide general comments, feedback, and suggestions.

### 2.3. Anatomic Visualization

**Figure 5:**
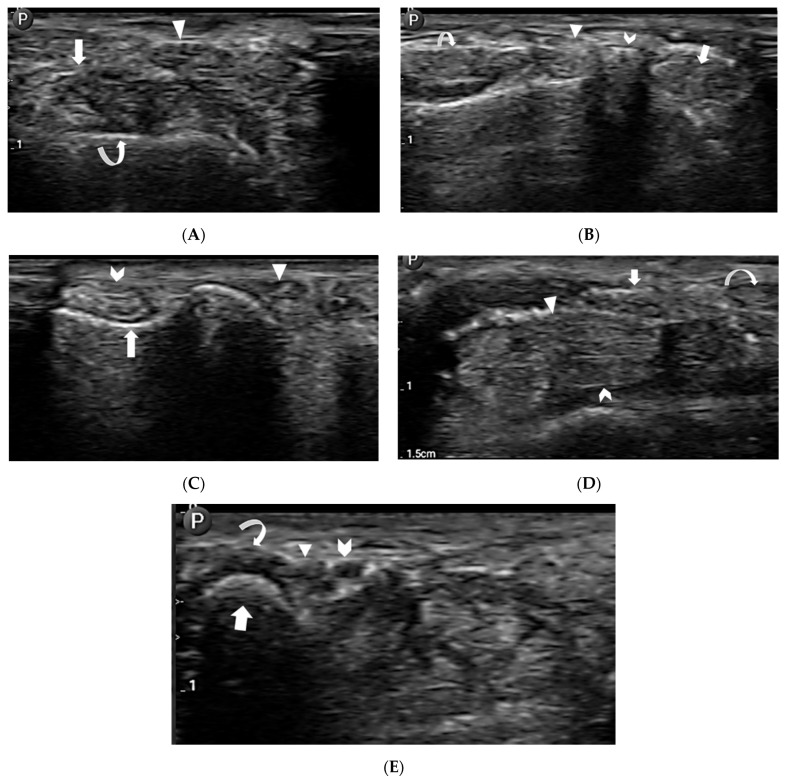
Telerobotic ultrasound images demonstrating: (**A**) the intersection area between the first extensor compartment (arrow) and third extensor compartment (arrow head). The cortex of the radial styloid process is also shown (curved arrow), (**B**) the second extensor compartment tendons (arrow), third extensor compartment (arrow head), fourth extensor compartment tendons (curved arrow), and Lister’s tubercle (chevron), (**C**) the fifth extensor compartment (arrow head), sixth extensor compartment (chevron), and distal ulnar groove for the extensor carpi ulnaris tendon (arrow), (**D**) carpal tunnel structures which includes the medial nerve (arrow), flexor digitorum superficialis (arrow head), and flexor digitorum profundus (chevron), and flexor carpi radialis tendon (curved arrow), and (**E**) Guyon’s canal structures which includes the ulnar artery (chevron), ulnar nerve (arrow head), flexor carpi ulnaris tendon (curved arrow), and triquetrum (arrow).

The trial aimed at examining a complex anatomical region: the wrist. Telerobotic ultrasound exams were conducted remotely, and targeted wrist structures included the following:

1. Extensor tendon compartments:
  - First extensor compartment—extensor pollicis brevis and abductor pollicis longus tendons (Figure 5A).
  - Second extensor compartment—extensor carpi radialis longus and brevis tendons (Figure 5B).
  - Third extensor compartment—extensor pollicis longus tendon (Figure 5A,B).
  - Fourth extensor compartment—extensor digitorum longus tendons (Figure 5B).
  - Fifth extensor compartment—extensor digiti minimi tendon (Figure 5C).
  - Sixth extensor compartment—extensor carpi ulnaris tendon (Figure 5C).
2. Flexor tendons:
  - Flexor carpi radialis tendon (Figure 5D).
  - Flexor carpi ulnaris tendon (Figure 5E).
  - Flexor digitorum superficialis tendons (Figure 5D).
  - Flexor digitorum profundus tendons (Figure 5D).
3. Median nerve (Figure 5D).
4. Guyon’s canal contents:
  - Ulnar artery (Figure 5E).
  - Ulnar nerve (Figure 5E).
5. Bony anatomy:
  - Radial styloid process (Figure 5A).
  - Lister’s tubercle (Figure 5B).
  - Triquetrum (Figure 5C).

## 3. Results

### 3.1. Anatomic Visualization

The results of the radiologist’s assessments of communication with participants, image quality, ergonomics, and convenience are shown in Table 1. All tendons and neurovascular structures were correctly evaluated, and the image quality scored 5 out of 5 on a Likert scale with no image distortion or artefact. The ability to remotely control ultrasound parameters including gain, zoom, depth, and focus allowed the radiologist to optimize image quality. The frequency of anisotropy was comparable to that of a conventional technique.

### 3.2. Radiologist’s Experience

Ergonomics and convenience scored 4–5 out of 5 on a Likert scale. Communication between the radiologist and participants scored 4–5 out of 5 on a Likert scale with the majority (67%) scoring 5 out of 5. The gamepad controller was noted to be an improvement, owing to increased comfort and intuitiveness compared to a joystick.

### 3.3. Participants’ Experience

Force feedback was available to the radiologist at the master station which provided the examiner with visual guidance on the force exerted on the wrists. Anecdotally, this resulted in better tolerance of the telerobotic ultrasound exam by the participants when compared with the previous trial. Participants rated their comfort experience as very comfortable (*n* = 4; 33%), comfortable (*n* = 5; 42%), and neither comfortable nor uncomfortable (*n* = 3; 25%). None of the participants complained about the examination being painful. Participants rated their communication experience with the radiologist as very good (*n* = 8; 67%), good (*n* = 3; 25%), and poor (*n* = 1; 8%). The participant who described the communication as poor provided feedback that the poor communication was due to the quiet microphone. Therefore, the different network configuration or delay did not significantly impair communication.

### 3.4. Delay and Time Measurement

The communication round-trip delay, or latency, was measured using a timer programmed into the control software. Data was successfully measured for all twelve trials. The comparison between the new VPN configuration and WLAN configuration can be seen in Table 2. The mean delay using the VPN and WLAN was found to be 26 and 28 ms, respectively.

Comparing the delay times, both network configurations behaved similarly to each other. The main difference was that the VPN configuration had delay spikes which significantly increased the maximum delay. These delay spikes were rare. Of the roughly 110,000 samples taken, only 17 were found to be over 400 ms. Therefore, nearly all of the data points will have little-to-no effect on performance [27]. If the user has some tolerance for the very rare delay spike, it may be acceptable. A different VPN service provider may also be more stable.

The unidirectional delay of the entire system was also measured using synchronized video recordings of the master and slave stations. This measures the entire delay from the controller input to the resulting motion of the robotic manipulator. The minimum and maximum system delays were found to be 67 and 634 ms, respectively. The delays within the 5th and 95th percentiles were found to be 131 and 403 ms. The mean and median delay were 249 and 250 ms. These values are consistent with previous measurements which found the mean delay to be 239 ms [26]. Note that the measured maximum for the system delay is smaller than the maximum network delay. This is because the delay spikes in the network occurred when the device was not moving. Thus, the network delay was not captured in the system delay measurement. As a result, the delay spikes did not affect performance in this trial. No significant difference was found between the delay in each degree of freedom.

**Figure 6:**
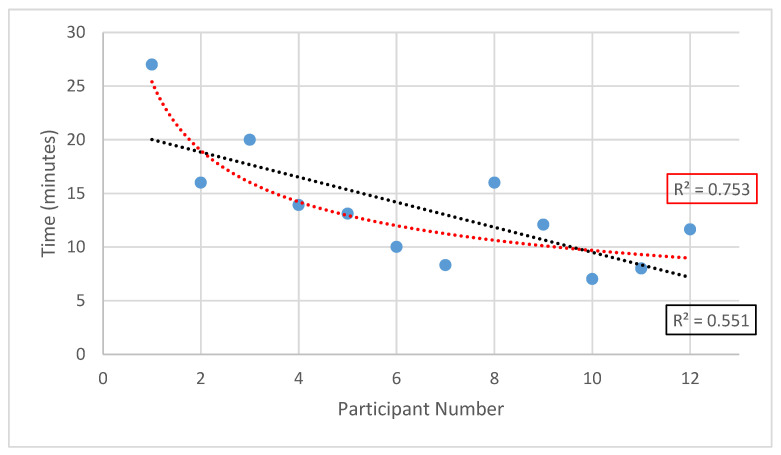
Combined examination time of both arms per participant versus participant number, across the trial; including linear trendline (black) and power trendline (red).

As the controller was changed from a joystick to a controller, the overall examination time was measured to assess the intuitiveness of the controller. The time was captured using video recording; the result can be seen in Figure 6. The radiologist showed a notable improvement over the trial as the scan duration improved from 27 min to a minimum of 7 min. Significant improvements occurred especially at the beginning of the trial as the radiologist became familiar with the controls. A break of at least an hour was taken after participants 4 and 8. However, the examination time did not noticeably change for subsequent participants, indicating retention in training. One factor which affected individual examination times was communication with the participant on how to position their arm for optimal imaging.

Conducting a statistical analysis of the examination times, a linear trendline exhibits an r-squared value of 0.551 while a power function exhibits an r-squared value of 0.753. Large improvements occurred, especially at the beginning of the trial as the radiologist became familiar with the controls.

## 4. Conclusions

Robots have shown significant promise in medicine, improving safety and accessibility for both patients and medical practitioners. This study focused on further development of a remote ultrasound robotic device for MSK imaging (MSK-TIM). Several improvements were made to the MSK device, including adding force feedback, inter-network control, and a better controller which improved the functionality of the device and allowed for more complex parts of the anatomy to be imaged. The images were considered to have satisfactory diagnostic quality with no artefacts or image distortion.

As a result of the technological advancements to the telerobotic system, there have been improvements in the radiologist’s and participants’ overall experience. This was demonstrated in a more comfortable scan experience with 75% of the participants describing their experience as either comfortable or very comfortable and 25% describing their experience as neither comfortable nor uncomfortable. There has been improved communication between the radiologist and participants with 11 out of the 12 participants describing their communication with the radiologist as either good or very good.

The gamepad controller on the master site was noted to be an ergonomic improvement over the joystick. In addition, the availability of remote access to ultrasound parameters such as depth, focus, and gain provided the radiologist with the ability to optimize image quality. These system improvements have resulted in a better and more intuitive experience for the radiologist. Considering the measured latency, it was found that implementing remote control via a VPN feasibly works for telesonography. The average network delay in the VPN configuration was found to be similar to the previous WLAN configuration. In summary, the modifications improved both the radiologist and patient experience with MSK-TIM.

The next step in this research is to enroll patients in a clinical trial to assess soft tissue pathology, with participants scanned by two radiologists and one sonographer. Future work should also consider alternative setups, especially considering that telemedicine can reach remote or resource-limited areas. These include connections over a mobile or satellite network with a variety of network conditions including variable speed and bandwidth. Finally, this research can be continued by investigating different upgrades to the existing MSK-TIM device. For example, different control schemes can be developed to mitigate the effects of delay. Additionally, a haptic force feedback system can be developed. Different methods of visual and haptic force feedback can be tested to measure their effectiveness in reducing examination and training time.

## Figures and Tables

**Table 1:** Radiologist and participant scores for the robotic tele-ultrasound system.

Radiologist’s Assessment					
	**1**	**2**	**3**	**4**	**5**
Communication	0 (0)	0 (0)	0 (0)	4 (33)	8 (67)
Image quality	0 (0)	0 (0)	0 (0)	0 (0)	12 (100)
Ergonomics	0 (0)	0 (0)	0 (0)	6 (50)	6 (50)
Convenience	0 (0)	0 (0)	0 (0)	4 (33)	8 (67)
Participants’ Assessment					
	**1**	**2**	**3**	**4**	**5**
Comfort	0 (0)	0 (0)	3 (25)	5 (42)	4 (33)
Communication	0 (0)	1 (8)	0 (0)	3 (25)	8 (67)

Likert scale from 1 to 5 where 1 represents inadequate performance and 5 represents perfect performance levels. Values are *n* (%).

**Table 2:** Comparing delay by network configuration.

Network Configuration		
	WLAN	VPN
5th Percentile (ms)	8	10
Median (ms)	11	13
Mean (ms)	28	26
95th Percentile (ms)	235	195
Maximum (ms)	285	3613

## Data Availability

Because of restrictions, data are only available on request. The data presented in this study are available on request from the corresponding author.
